# Immunotherapy for Squamous Esophageal Cancer: A Review

**DOI:** 10.3390/jpm12060862

**Published:** 2022-05-25

**Authors:** Angelica Petrillo, Elizabeth C. Smyth

**Affiliations:** 1 Medical Oncology Unit, Ospedale del Mare, 80147 Naples, Italy; 2 Cambridge University Hospitals NHS Foundation Trust, Hill’s Road, Cambridge CB2 0QQ, UK; elizabeth.smyth2@nhs.net

**Keywords:** adjuvant treatment, nivolumab, first-line treatment, neoadjuvant, immune checkpoint inhibitors, biomarkers

## Abstract

Esophageal squamous cell carcinoma (ESCC) is a rare gastrointestinal tumour with high mortality. A multimodality treatment based on chemoradiotherapy followed by surgery is the standard of care in the case of non-metastatic disease; chemotherapy has historically been the gold standard in the metastatic setting. However, the rate of relapse after curative treatment is high and the prognosis of ESCC is poor. In this context, immunotherapy is a novel and intriguing chance to improve survival. Therefore, in this narrative review, we depict the current scenario in the field of immunotherapy for ESCC according to the stage of disease and alongside the discussion of promising biomarkers and future perspectives. The Checkmate-577 trial showed that nivolumab is the best option as adjuvant treatment in patients with non-metastatic ESCC and residual disease after a multimodality approach. In the metastatic setting, nivolumab, pembrolizumab, camrelizumab, sintilimab and toripalimab improved survival outcomes as a first-line treatment in addition to chemotherapy. In the second-line, nivolumab, pembrolizumab, camrelizumab and tislelizumab showed positive results, with differences according to the subgroups, agents and study population included in the trials. Then, the finding of valid molecular biomarkers is crucial in selecting patients for immunotherapy.

## 1. Introduction

Oesophagal cancer (EC) is the seventh most common tumour in incidence worldwide, accounting for 604,100 new cases in 2020 (3.1% of new cancer cases); it has one of the higher mortality rates, with 544,076 deaths in 2020 [1].

However, today it is known that EC should not be considered and treated as a single entity. In fact, we can historically distinguish two main types of EC according to the histology: squamous cell carcinoma (SCC) and adenocarcinoma (EAC). SCC is mainly detected in the upper or middle oesophagus, especially in patients with smoke and use alcohol in Eastern Europe or Asia. On the other side, EAC is mainly located in the distal oesophagus and is linked with obesity or with the presence of oesophago-gastric reflux disease and Barrett’s oesophagus. EAC is the most frequent histotype in Europe and North America. Recently, the Cancer Genome Atlas (TCGA) classification confirmed that EAC and SCC are two different entities also from a molecular point of view [2]. Therefore, since EAC and SCC differ from histological, molecular, anatomical and histological points of view, we should consider a different therapeutic approach for each of them, also according to the stage of disease [3,4]. 

However, despite all the available treatments, the survival for these patients remains poor, especially due to the late diagnosis and the high rate of relapses even in the case of localized disease. In particular, among all ECs, SCC has the worst prognosis, especially in the case of metastatic disease. Additionally, patients affected by oesophageal SCC (ESCC) are complex, also due to the presence of concomitant diseases in most of them, their social profile, the need for a continuous nutritional assessment and the palliation of symptoms such as dysphagia or bleeding. Thus, they should be managed in the context of a multidisciplinary team dedicated to upper gastrointestinal tumours and in high-volume centres. 

Then, there is a lack of biomarkers able to individualize treatment for these patients. Therefore, the identification of new targets and/or biomarkers is greatly needed in order to tailor the treatments and improve the outcomes.

In this context, immunotherapy is becoming a novel and intriguing prospect in the field of ESCC. In fact, while the results of the landmark clinical trials have led to the approval and use of immune checkpoint inhibitors (ICIs) in many kinds of tumours over the last decades [5], identifying them as a huge chance for improving survival in those patients, their use in EC (both SCC and EAC) is recent and mainly still confined to clinical trials. 

Based on this background, this narrative review aims to depict the current scenario in the field of immunotherapy for ESCC. In particular, we provide an overview of the role of ICIs in this field, according to the stage of disease, alongside a discussion of the promising biomarkers and future perspectives. 

## 2. Non-Metastatic ESCC: Adjuvant and Neoadjuvant Settings

According to international guidelines [3,4], multidisciplinary treatment is considered the standard of care in case of non-metastatic ESCC. In fact, this approach can reduce the rate of relapse, which was high after up-front curative surgery, and improve outcomes by treating the micrometastases. Doublet chemotherapy based on fluoropyrimidine and platin or according to the CROSS schedule [6,7] is the main therapeutic option for ESCC as definitive treatment in case of cervical tumour or unresectable disease; in the other cases, a multimodality approach with chemoradiotherapy followed by surgery is the best option.

Moving from the standard of care, the research is now focusing on the role of immunotherapy in this field. In particular, the anti-programmed death 1 (PD-1) drugs, such as pembrolizumab, nivolumab, camrelizumab and sintilimab, are the main agents tested in the non-metastatic ESCC. 

Recently, the data from the randomized and global phase III Checkmate-577 trial are the most important findings in the adjuvant setting [8]. The trial included 794 patients with resectable (stage II-III) oesophageal (both ESCC and EAC) and gastroesophageal junctional adenocarcinoma (GEJA) who received neoadjuvant treatment with chemoradiotherapy. Among them, the patients who did not achieve a pathological complete response (pCR) on the surgical specimen after R0 resection were randomized 2:1 to receive adjuvant treatment with nivolumab or placebo; the patients were not selected for programmed death ligand 1 (PD-L1) expression. The trial met its primary endpoint, showing a doubling in the median disease-free survival (DFS) in the experimental arm (22.4 versus 11 months in nivolumab and control arm, respectively; hazard ratio (HR): 0.69, *p* < 0.001). The treatment was well tolerated, showing grade 3–4 adverse events (AE) in 13% of patients versus 6% in the placebo group. Of note, in the Checkmate- 577 trial, the study population was heterogeneous, with 13.3% of patients from Asian countries, 60% of tumours located in the oesophagus and 40% in the gastroesophageal junction. Additionally, it included 71% of adenocarcinoma and 29% of ESCC. In this regard, nivolumab improved the median DFS across tumour’s histotypes with higher benefit in the case of ESCC (ESCC: 29.7 versus 11.1 months, HR: 0.61; adenocarcinoma, both oesophageal and junctional: 19.4 versus 11.1 months, HR: 0.75). Of note, there was a 13% reduction in disease recurrence or death in patients with GEJA (HR: 0.87). Lastly, we should take into account that the trial evaluated PD-L1 expression firstly by tumour proportion score (TPS) and then by combined positive score (CPS) as part of the post hoc analysis. Therefore, the patients were stratified at randomization according to PD-L1 TPS expression (<1%:83.8% of patients and ≥1%:16.2%). However, when the slides were revised according to PD-L1 CPS, 57% of patients in the nivolumab arm and 54% of patients in the placebo arm had CPS ≥ 5 (PD-L1 positive). The post hoc analysis on PD-L1 positive patients showed a clear benefit in this subgroup. However, as already mentioned, it was a post hoc analysis and the patients were not stratified according to CPS. Therefore, we could consider nivolumab as the best option in those patients and PD-L1 as a dynamic biomarker in the adjuvant setting.

Finally, in the case of patients with locally advanced ESCC who cannot undergo surgery after neoadjuvant treatment, the phase III SKYSCRAPER-07 (NCT04543617) is comparing tiragolumab (anti- T cell immunoreceptor with Ig and ITIM domains (TIGIT) agent) plus atezolizumab (anti-PD-L1 agent) versus atezolizumab versus placebo as maintenance treatment in patients who have not progressed after chemoradiotherapy [9].

Regarding the neoadjuvant setting, the evidence of using ICIs in oesophageal cancer is scarce [10]. In particular, Wu Z et al. [11] recently evaluated the safety and efficacy by using a combination of immunotherapy (camrelizumab, pembrolizumab or sintilimab) and chemotherapy (docetaxel or paclitaxel in addition to a platinum doublet) as neoadjuvant treatment for 38 ESCC (stage II-IVb). The analysis showed major radiological responses in 42.11% of patients, and a 92.11% of R0 resection rate, without increasing surgical complications. However, the retrospective design, the inclusion of different stages of disease (heterogeneity) and the small sample size of the analysis do not allow us to draw conclusions on the benefit of using immunotherapy in this setting. 

Then, Shen D et al. [12] assessed the safety and feasibility of a combination of immunotherapy (nivolumab, pembrolizumab or camrelizumab) and chemotherapy (carboplatin and nab-paclitaxel) as neoadjuvant treatment for 28 ESCC. The analysis showed pCR in 33.3% of patients, major radiological responses in 85.7% of patients, and 96.3% of R0 resection rate without increasing surgical complications. However, the small sample size and the use of unconventional neoadjuvant chemotherapy are important limitations of this analysis. 

In this setting, the NCT04280822 and the Keynote-975 (NCT04210115) trials are currently ongoing. In particular, the Chinese phase III randomized and controlled NCT04280822 study is currently evaluating the efficacy of adding toripalimab (JS001, a novel anti-PD-1 antibody) to neoadjuvant chemotherapy for resectable ESCC followed by surgery and adjuvant treatment with toripalimab or placebo. The global phase III Keynote-975 trial is evaluating the efficacy of adding pembrolizumab to definitive chemoradiotherapy in locally advanced ESCC, EAC or GEJA, first-line metastatic cervical ESCC or oligometastatic upper thoracic tumours (supraclavicular lymph nodes metastases only) [13]. 

In conclusion, adjuvant treatment with nivolumab is currently the best option in patients with resected oesophageal (both EAC and ESCC) or GEJA and residual pathological disease after neoadjuvant treatment. On the other hand, further prospective evaluations are needed in order to confirm the role of immunotherapy in the neoadjuvant setting for ESCC. Additionally, the research of potential biomarkers might be useful to select patients as candidates for immunotherapy.

## 3. Metastatic Disease

### 3.1. First-Line Treatment

Doublet chemotherapy based on platin and fluoropyrimidine/taxanes is the standard of care in the first-line treatment for metastatic ESCC [3]. However, the survival outcomes of using those agents are still disappointing with median overall survival (OS) of 8–10 months.

The use of immunotherapy in combination with chemotherapy has recently shown exciting results, changing the therapeutic scenario in this setting. In particular, the efficacy of anti PD-1 drugs was explored in the randomized phase III Keynote-590 [14,15], ESCORT-1 [16], Checkmate 648 [17], ORIENT-15 [18] and JUPITER-06 [19] trials (Table 1). Of note, in the Checkmate 648 trial [17], nivolumab was tested also in combination with ipilimumab (anti-CTLA4 agent).

The global Keynote-590 trial [14] included 749 patients from 131 centres in 26 countries affected by metastatic oesophageal and gastroesophageal tumours (Siewert I) who did not receive any previous treatment for metastatic disease, regardless of PD-L1 status. The patients were randomized to receive standard first-line chemotherapy (cisplatin and 5-fluorouracil) alone or in combination with pembrolizumab (200 mg flat dose every three weeks for a maximum of 35 cycles) until progression or unacceptable toxicity. The recently published interim analysis showed that the addition of pembrolizumab to chemotherapy improved the outcomes in all the subgroups. In detail, there was an improvement of 5 months in OS in patients with ESCC and PD-L1 ≥ 10, 3 months in patients with ESCC, 4 months in patients with PD-L1 ≥ 10 and almost 3 months in the entire study population (see Table 1 for additional details). Same positive results were reported for progression-free survival (PFS). However, in the exploratory analysis there was no benefit to adding pembrolizumab to chemotherapy in patients with PD-L1 < 10. The combined treatment had a manageable safety profile; however, 72% of patients in the experimental arm had a grade 3–4 AE versus 68% in the control arm. The most frequent grade 3–4 AE in the combination arm were decreased neutrophil count (23%), neutropenia (14%), anaemia (12%) and nausea (7%). Additionally, 26% of patients in the pembrolizumab arm versus 12% in the placebo arm had immune-related AE (grade 3: 7% versus 2%); among them, hypothyroidism (11%), pneumonitis (6%) and hyperthyroidism (6%) were the most common. Of note, PD-L1 was assessed by using CPS and patients were not stratified by PD-L1 expression. Additionally, it is important to highlight that the trial included 73% of patients affected by ESCC, whereas 27% had adenocarcinomas (EAC or Siewert I GEJA) and 53% of patients were from Asian countries. In this regard, the trial showed no significant benefit in OS for patients with adenocarcinoma or from non-Asian countries, even if there were positive results in PFS in those subgroups.

Those data were recently confirmed after a longer follow-up [15]. Interestingly, in the updated analysis, 20% of patients in the experimental arm versus 6% in the control arm have maintained the response for a long period (≥24 months). Based on those results, pembrolizumab was approved by authorities in this setting for all patients (regardless of PD-L1 status) in the USA and for patients with PD-L1 ≥ 10 in Europe.

The recently published ESCORT-1 trial [16] randomized 595 Chinese patients with naïve metastatic ESCC to receive chemotherapy (cisplatin 75 mg/mq and paclitaxel 175 mg/mq every three weeks for six cycles) as first-line treatment, alone or in combination with camrelizumab (200 mg every three weeks until disease progression). The trial met its primary endpoints; the addition of camrelizumab to standard chemotherapy improved OS (median OS: 15.3 versus 12 months in the experimental and control arm, respectively; HR: 0.70, *p* = 0.001) and PFS (median PFS: 6.9 versus 5.6 months, HR: 0.56, *p* = 0.001) in those patients with a manageable safety profile (grade 3–4 AE: 63.4% versus 67.7% in the combination and control arm, respectively). The most frequent grade 3–4 AEs in the combination arm were decreased neutrophil count (39.9%), white cell count decrease (24.2%) and anaemia (17.4%). However, we should consider that the trial was totally conducted in China and, thus, included only Asian patients. Additionally, the benefit in survival (both OS, 3 months improvement, and PFS, 1.3 months improvement) has quite low consistency if we consider the setting (first-line treatment). Finally, there was no selection according to PD-L1 status.

The global open-label Checkmate 648 trial [17] randomized 970 patients (Asian and western) with previously untreated metastatic ESCC to receive nivolumab (240 mg flat dose every two weeks) plus chemotherapy (5-fluorouracil plus cisplatin every four weeks), nivolumab (3 mg/kg every two weeks) and ipilimumab (1 mg/kg every six weeks), or chemotherapy alone, regardless the PD-L1 expression. The paper in extenso reporting the final results was recently published. The experimental arms showed improved OS when compared to the control arm in the entire study population (OS: 13.2, 12.7 and 10.7 months in the nivolumab plus chemotherapy (HR: 0.74), nivolumab plus ipilimumab (HR: 0.78) and chemotherapy arm, respectively). The benefit in OS was greater in patients with PD-L1 ≥ 1% (OS: 15.4, 13.7 and 9.1 months in the nivolumab plus chemotherapy (HR: 0.54), nivolumab plus ipilimumab (HR: 0.64) and chemotherapy arm, respectively) as well as in PFS for nivolumab plus the chemotherapy arm (PFS: HR: 0.65, *p* = 0.00023), whereas the nivolumab plus ipilimumab arm did not meet the PFS endpoint. The experimental arms showed an improvement in objective response rates (ORR) if compared with the control arm, especially in patients with PD-L1 ≥ 1% (53% in nivolumab plus chemotherapy, 35% in nivolumab plus ipilimumab and 20% in the chemotherapy arm) and with long duration of responses. Regarding the safety profile, grade 3–4 AEs were reported in 47% of the nivolumab plus chemotherapy arm, 32% of the nivolumab plus ipilimumab arm and 36% of the chemotherapy arm. It is important to note that PD-L1 was assessed by TPS, even if a pre-planned analysis for CPS was done too. Additionally, the Kaplan Meyer plot showed a crossing of the curves in the nivolumab plus ipilimumab arm at almost 6.5 months, underlining the need to select patients as candidates for this strategy. The authors concluded that the combination arms (nivolumab plus chemotherapy and nivolumab plus ipilimumab) could be considered a new therapeutic option in the first-line treatment for metastatic ESCC with a good safety profile. However, this assumption should be translated with caution in clinical practice, considering the results of the two experimental arms separately. In fact, if the addition of nivolumab to chemotherapy showed a positive impact on survival, the use of nivolumab and ipilimumab alone showed ORR similar to chemotherapy in the entire population, but worse PFS, AE comparable to chemotherapy and crossing of the curves. Therefore, further data after a longer follow-up are needed for this arm in order to evaluate the magnitude of benefit in this setting.

Finally, the first results of the ORIENT-15 [18] and JUPITER-06 [19] trials were recently presented. The ORIENT-15 trial is a phase III and global study that has investigated the role of adding sintilimab to chemotherapy as a first-line treatment for metastatic ESCC [18]. The trial randomized 659 patients to receive sintilimab (200 mg flat dose every three weeks for up to 24 months) plus chemotherapy (paclitaxel 175 mg/mq and cisplatin 75 mg/mq every three weeks or cisplatin 75 mg/mq and 5- fluorouracil 800 mg/mq on day 1–5 every three weeks for six cycles) or placebo plus chemotherapy. The patients were included regardless of PD-L1 status; however, the trial assessed PD-L1 positivity by both TPS and CPS scores. The results showed that the addition of sintilimab to chemotherapy was superior in terms of OS (16.7 versus 12.5 months, HR: 0.628, *p* < 0.0001) and PFS (7.2 versus 5.7 months, HR: 0.558, *p* < 0.0001) in the entire study population. However, patients with PD-L1 CPS ≥10 had the greatest benefit (median OS: 17.2 versus 13.6 months, HR: 0.638, *p* = 0.0018; median PFS: 8.3 versus 6.4 months, HR: 0.58, *p* < 0.0001). The same results were shown in terms of ORR for the experimental arm (entire population: 75.5% versus 56.9%; PD-L1 CPS ≥10:78.7% versus 57.5%) with a comparable safety profile (grade 3–5 AE: 59.9% versus 54.5%). Of note, 97.1% of the study population was from China. The authors concluded that sintilimab plus chemotherapy could be considered a new therapeutic option in the first-line treatment for metastatic ESCC. However, the final results and the paper in extenso are still awaited

The JUPITER-06 trial is a phase III trial that is evaluating the efficacy and safety of toripalimab as a first-line treatment for metastatic ESCC in an Asian population [18]. The trial randomized 514 patients to receive toripalimab (240 mg flat dose every three weeks) plus chemotherapy (paclitaxel 175 mg/mq and cisplatin 75 mg/mq every three weeks for six cycles) or chemotherapy alone, followed by toripalimab as a maintenance treatment or placebo, regardless of PD-L1 status. The first results showed that the addition of toripalimab to chemotherapy has improved OS and PFS in all the subgroups (median OS: 17 versus 11 months, HR: 0.58, *p* = 0.00037; median PFS: HR: 0.58, *p* < 0.00001) with comparable safety profile to chemotherapy alone (grade 3–5 AE: 73.2% versus 70.0%). However, the rate of fatal events was higher if indirectly compared to other trials in this field (8.2%). Furthermore, in this case, the author concluded with a positive statement regarding the efficacy of the combination arm. However, final results are awaited.

For the ongoing trials in this setting, see Table 1.

In conclusion, immunotherapy has changed the standard of care in the first-line treatment for metastatic ESCC and additional novel agents are currently being tested in this field. Nevertheless, the lack of direct comparison in this setting as well as the heterogeneity of the study population and methods (e.g., PD-L1 score and assessment) do not allow to select a single best choice of anti-PD-1 inhibitor as the first-line treatment at the time of writing. In the non-Asian population, pembrolizumab plus chemotherapy and nivolumab plus chemotherapy are the two options with the most consistent data up to date; however, the first is recommended in Europe only in patients with PD-L1 ≥ 10. In an Asian population, the addition of camrelizumab, sintilimab or toripalimab to chemotherapy could be good therapeutic options in this setting. 

### 3.2. Second and Later Lines Treatment 

The ICIs in ESCC were historically tested in the later lines of treatment for metastatic disease. According to international guidelines [3,4], patients who maintain a good performance status after progression to chemotherapy for the metastatic disease should be candidates to receive another line of treatment. In this regard, if chemotherapy based on taxanes or irinotecan schedules has been considered the standard of care over many years, today we recognize immunotherapy as the best option for these patients [3,4]. 

The anti-PD1 nivolumab, pembrolizumab, camrelizumab and tislelizumab are the ICIs tested as a single agent in the later lines for ESCC and the phase III ATTRACTION-3 [21], Keynote-181 [22], ESCORT [23] and RATIONALE 302 [24] trials are the landmark studies in this field (Table 2).

The ATTRACTION-3 trial [21] is a multicentre, global and phase III trial in which 419 patients with advanced or metastatic ESCC were randomized to receive nivolumab (240 mg every two weeks until disease progression or unacceptable toxicity) or chemotherapy (paclitaxel 100 mg/m^2^ every week for six weeks on and one week off or docetaxel 75 mg/m^2^ every 3 weeks at investigator’s choice) as a second-line treatment after failure of a fluoropyrimidine-based treatment. The patients were included regardless of PD-L1 status. Of note, although the trials were global, only 4% of the study population (18 patients) were not from Asia. The trial met its primary endpoint, showing superiority in OS for nivolumab when compared to chemotherapy (median OS: 10.9 versus 8.4 months, respectively; HR: 0.77, *p* = 0.019). As in previous experiences in other kinds of tumours [25,26], nivolumab failed to improve PFS (1.7 versus 3.4 months in the chemotherapy arm, HR: 1.08). However, the crossing of the curves in the Kaplan–Mayer plot clearly showed that the selection of patients might be crucial in order to improve the outcomes in this setting. For instance, in the “responders” the curves tend to have a plateau, showing the maintenance of response for a long period. In detail, 19% of patients in the nivolumab and 22% in the chemotherapy arm showed an objective response; in this case, the duration of response was improved in the nivolumab group (6.9 versus 3.9 months), confirming that the patients who respond maintain the response for a long time. The disease control rates (DCR) were 37% versus 63% in the experimental and control arm, respectively. The safety profile was favourable for the nivolumab group (18% versus 63% of grade 3–4 related AE). Thus, based on those results, nivolumab received the European commission approval for ESCC in November 2020 [27]; it is considered the new standard of care in the second-line treatment for metastatic ESCC after a fluoropyrimidine-based chemotherapy, regardless of PD-L1 status. 

**Table 2:** Immune checkpoint inhibitors in the second and later lines setting for metastatic oesophageal squamous cell cancer: landmark phase III and main ongoing trials.

Trial/ NCT Number	Phase/Line/State of the Trial *	Ethnicity: Asian/Western (%)	Tumour Histology and Primary Site Location	Regimens	Patients (n)
Landmark Trials					
ATTRACTION-3 [21]	III/2nd line	Asian (96%) Western (4%)	ESCC (100%); NR	Nivolumab Chemotherapy (docetaxel or paclitaxel)	210 209
Keynote-181 [22]	III/2nd line	Asian (38.7%) Western (61.3%)	ESCC (64.2%) EAC and GEJA Siewert I (35.8%)	Pembrolizumab Chemotherapy (docetaxel, paclitaxel or irinotecan)	314 314
ESCORT [23]	III/2nd line	Asian (100%)	ESCC (100%); NR	Camrelizumab Chemotherapy (docetaxel or irinotecan)	228 220
RATIONAL 302 [24]	III/2nd line	Asian (79%) Western (21%)	ESCC (100%); NR	Tislelizumab Chemotherapy (docetaxel, paclitaxel or irinotecan)	256 256
Ongoing trials					
BEAR (NCT04839471)	II/≥ 2nd line/Enrolling by invitation	Asian	ESCC; NR	BI-754091 plus afatinib	NA
RAMONA [28] (NCT03416244)	II/2nd line/active, not recruiting	Western, elderly	ESCC; NR	Nivolumab Nivolumab + ipilimumab	NA

* state of the trial was shown only for the ongoing trials. Abbreviations: ESCC: oesophageal squamous cell carcinoma; NR: not reported; EAC: oesophageal adenocarcinoma; GEJA: gastroesophageal junctional adenocarcinoma; NE: not evaluable.

The Keynote-181 trial is an open-label, multicentre and global phase III study that randomized patients with advanced or metastatic oesophageal cancer to receive pembrolizumab (200 mg flat dose every three weeks) or chemotherapy (at investigator’s choice: paclitaxel 80–100 mg/mq on day 1, 8 and 15 every 28 days, irinotecan 180 mg/mq every two weeks or docetaxel 75 mg/mq every three weeks) as a second-line treatment [22]. The treatments were administered until disease progression or unacceptable toxicity. The trial included 628 patients with either ESCC or EAC as well as Siewert I epidermal growth factor receptor 2 (HER-2) negative GEJA. It showed that pembrolizumab improved median OS compared to the control arm in the entire population with PD-L1 expression ≥ 10, according to CPS (9.3 versus 6.7 months, HR: 0.69, *p* = 0.0074) and in patients with ESCC (8.2 versus 7.1 months, HR: 0.78, *p*: 0.0095), especially in the Asian population. However, the result in the ESCC cohort did not reach the preplanned boundaries and, thus, the trial did not meet the co-primary endpoint in median OS. Additionally, the trial showed no difference in median OS in the entire population, regardless of PD-L1 and histology (7.1 versus 7.1 months, HR: 0.89, *p* = 0.056) as well as no benefit in terms of median PFS (PD-L1 ≥ 10 subgroup: 2.6 versus 3.0 months in the experimental and control arm, respectively; ESCC subgroup: 2.2 versus 3.1 months; entire population: 2.1 versus 3.4 months). Regarding the responses, pembrolizumab improved the ORR in the PD-L1 ≥ 10 (regardless of the histology, 21.5% versus 6.1%) and ESCC subgroups (16.7 versus 7.4%), whereas no benefit in responses was showed in PD-L1 < 10 subgroup. Then, the treatment was well tolerated, showing 18% grade 3–5 AEs in the experimental arm versus 40.9% in the chemotherapy arm. However, the EMA in the first evaluation did not approve the use of pembrolizumab in this setting due to the low improvement in the outcomes with no clear gain of the benefit for the treatment over the risks. 

The ESCORT trial is a Chinese, multicentre and open-label phase III trial, which randomized 457 patients with advanced or metastatic ESCC to receive camrelizumab (200 mg flat dose every two weeks) or chemotherapy (docetaxel 75 mg/mq every three weeks or irinotecan 180 mg/mq every two weeks) as a second-line treatment [23]. The treatments were administered until disease progression or unacceptable toxicity. The patients were included regardless of PD-L1 status. Camrelizumab showed to improve median OS in the study population (8.3 versus 6.2 months, HR: 0.71, *p* = 0.001) and in all the subgroups with a good safety profile (grade 3–5 AEs: 19% versus 39%). However, patients with PD-L1 positive tumours (≥1% according to TPS) had the greatest benefit from treatment. No difference in median PFS was reported in the two groups (1.9 versus 1.9 months, respectively, HR: 0.69, *p* = 0.00063), whereas the ORR and DCR were in favour of the experimental arm (ORR: 20.2% versus 6.4% in camrelizumab and chemotherapy arm, respectively; DCR: 44.7% versus 34.5%, respectively). Therefore, camrelizumab is a good therapeutic option in the second-line treatment for ESCC, regardless of the PD-L1 expression. However, we should consider that those results are referred only to a Chinese population; of note, seven patients in the experimental arm (3% versus 1% in the chemotherapy arm) died (one for hepatic failure, one for enterocolitis, one for pneumonia, one for myocarditis and three for unknown reasons).

The RATIONALE 302 trial is a global, multicentre and phase III trial, that randomized 512 patients with advanced or metastatic ESCC to receive tislelizumab (200 mg flat dose every three weeks) or chemotherapy (docetaxel, paclitaxel or irinotecan) as a second-line treatment [24]. The patients were included regardless of PD-L1 status; however, subgroup analysis according to PD-L1 status per visual CPS (vCPS) was performed. The recently presented preliminary results showed that the trial met its primary endpoint. In fact, there was a 2.3 months improvement in median OS in the experimental arm (median OS: 8.6 versus 6.3 months in the control arm, HR: 0.70, *p* = 0.0001) in the entire study population across the subgroups. The benefit was greater in the PD-L1 vCPS ≥ 10 subgroup (157 patient (30% of the study population); median OS: 10.3 versus 6.8 months; HR: 0.54, *p* = 0.0006). Additionally, tislelizumab showed a good safety profile (grade 3–5 AE: 46% versus 68%) and a higher rate of responses if compared to chemotherapy (ORR: 20.3% versus 9.8%, respectively). Therefore, tislelizumab might be a potential therapeutic option in this setting. Nevertheless, at the time of writing the results were presented only in the abstract form.

Lastly, see Table 2 for the ongoing trials in this setting.

Despite those positive results, no direct comparisons between ICIs in the second-line setting for ESCC exist and only indirect comparisons are available up to date (Table 3). In this regard, Zhou YX et al. [29] showed an indirect similar survival benefit among nivolumab, pembrolizumab and camrelizumab, even if nivolumab was better in patients with poorer performance status, whereas camrelizumab (first) and pembrolizumab (second) showed better PFS and ORR. However, camrelizumab was linked with higher toxicity rates. Nevertheless, due to the indirect nature of this comparison, further prospective evaluations are needed before doing general recommendations in this regard.

In conclusion, today an ICI-based treatment is the best option in the second-line treatment for patients affected by metastatic ESCC who did not receive immunotherapy in the first line. Otherwise, in the case of patients who already had ICIs in the first line, chemotherapy remains the standard of care at the time of writing. However, taking into account that eventually more patients will be a candidate in the next future to receive a first-line treatment based on immunotherapy, further trials assessing the efficacy of ICIs in pretreated patients are needed.

## 4. Molecular Biomarkers for Immunotherapy

Considering the poor prognosis and the biological behaviour of ESCC, especially in case of metastatic disease, the choice of the best therapeutic algorithm is crucial for these patients. In this process, the selection of patients to candidate to immunotherapy is one of the most important phases. In fact, the results of the landmark trials [13,14,15,16,17,18,21,22,23,24] suggest that there is a group of patients, so-called “responders”, which benefit more from immunotherapy. On the contrary, the trials showed that a group of patients does not benefit at all from immunotherapy. Thus, the use of ICIs in those patients might lead to a worsening of conditions, also linked with the time to respond, which is longer in the case of immunotherapy, or the hyperprogression. Therefore, the role of predictive as well as prognostic molecular biomarkers has been worth investigating during the last years in order to help in the selection of patients as candidates for immunotherapy.

Among them, PD-L1 expression is the most important. However, the landmark trials in this field showed a huge heterogeneity in the PD-L1 assessment, score and cut-off, which lead to a lack of standardization (Table 4). Nevertheless, taking into account those limitations, the role of PD-L1 as predictive and/or prognostic biomarkers has been explored. In this regard, a recently published systematic review and metanalysis from Leone AG et al. reported the data from 5257 patients included in 10 randomized controlled trials of immunotherapy for advanced ESCC [30]. In this analysis, immunotherapy showed to improve survival outcomes (HR: 0.71) regardless of the country (Asian versus non-Asian) and the line of treatment (first versus subsequent lines). Immunotherapy improved PFS and ORR too, with HR: 0.78 and odd ratio: 1.5, respectively. The analysis showed that the benefit in ESCC was higher in the case of PD-L1 CPS ≥ 10 (HR: 0.60 versus 0.83 in PD-L1 < 10, *p* = 0.009), suggesting a promising role for PD-L1 as molecular biomarkers in ESCC. However, the heterogeneity in the PD-L1 assessment in the landmark trials requires further prospective studies in order to validate the PD-L1 score to refer to in the clinical practice. This issue is of high interest if we consider that regulatory approval for using ICIs in clinical practice is often restricted to some subgroups of patients according to PD-L1 expression.

Then, the researchers are focusing on the finding of prognostic factors in ESCC, eventually matched in prognostic scores or signatures. In this regard, Yao J et al. recently developed a prognostic gene signature by analyzing 95 samples from the TCGA ESCC cohort [31]. The study showed that a 10-gene signature (C1QA, C1QB, C1QC, CD86, C3AR1, CSF1R, ITGB2, LCP2, SPI1, and TYROBP) was linked to M2 macrophage in the tumour microenvironment (TME) and poor prognosis in ESCC that expresses those genes (HR: 2.104, *p* = 0.001). Additionally, the analysis showed that C1QA, C3AR1, LCP2, SPI1, and TYROBP were up-regulated in tumours with the worst prognosis. However, how those findings might identify potential targets for treatment in ESCC and how the gene signature might work as biomarkers in ESCC treated by immunotherapy has yet to be defined. In this regard, Cao K et al. evaluated the prognostic role of immune-related gene signatures in ESCC and EAC [32]. The authors identified 19 genes for ESCC and 17 for EAC; they included 75 samples from ESCC ad 78 from EAC. The analysis showed a link between the immune-related gene signature, the composition of TME and response to ICI in both histotypes, even if with their own peculiarities. However, further prospective studies are needed in order to validate and use the signature as prognostic biomarkers for ESCC treated with immunotherapy.

Lastly, even if the data about the role of circulating tumour DNA as predictive and prognostic biomarkers have improved also in the ESCC field [33]; unfortunately, no data are available concerning immunotherapy at the time of writing. Therefore, further evaluation, eventually as an exploratory analysis from the landmark phase III trials, is awaited in order to clarify this issue.

## 5. Conclusions and Future Perspective

ESCC is a complex disease with a high rate of relapse even in the case of non-metastatic tumour and poor prognosis. Chemotherapy, alone or in combination with radiotherapy as part of a multimodality approach, has been the mainstream in the treatment of ESCC over many decades; however, it gained disappointing improvements in the survival of those patients. Indeed, the majority of patients show worsening clinical conditions during the therapeutic journey, and only a few patients maintain a good performance status, which allows us to put them forward to receive a later line treatment.

In this “hard-to-manage” field, the development of immunotherapy is a light at the end of a tunnel in ESCC and positive results are coming from the landmark trials [8,14,15,16,17,18,19,21,22,23,24]. In particular, in patients with resectable ESCC and a high risk of relapse after neoadjuvant treatment followed by surgery, adjuvant treatment with nivolumab is currently the best option [8], which will change the clinical practice after authorities’ approval.

**Figure 1:**
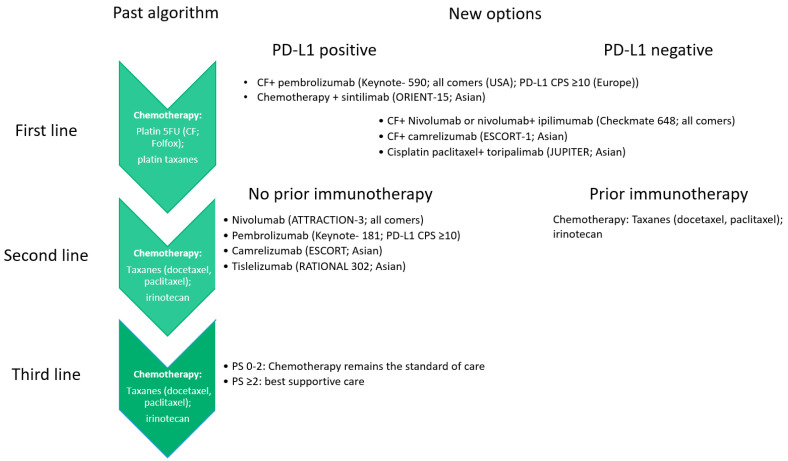
How immunotherapy has changed the treatment algorithm for metastatic ESCC. Abbreviations: 5FU: fluorouracil; CF: cisplatin, fluorouracil; PS: performance status according to ECOG scale; PD-L1: programmed death ligand-1; CPS: combined positive score.

Regarding the metastatic setting, immunotherapy has changed the treatment algorithm for ESCC (Figure 1). In particular, taking into account the lack of direct comparisons between different ICIs as well as the strength and limitations of the landmark trials [14,15,16,17,18,19,21,22,23,24], nivolumab and pembrolizumab are the main therapeutic options in addition to chemotherapy in the first-line setting or as a single agent in the second-line setting for ESCC. Nevertheless, if nivolumab showed to clearly improve the outcomes in those patients regardless of PD-L1 status (all comers), pembrolizumab should be reserved for patients with PD-L1 CPS ≥10. Then, camrelizumab, tislelizumab, sinlitimab and toripalimab might be promising options in Asian patients.

However, is important to underline that some cited trials have included both ESCC and EAC as well as GEJA Siewert I, which are known to be completely different entities. Additionally, there is still a debate regarding the influence of ethnicity in the responses, due to the peculiarities of ESCC in Asian versus non-Asian patients and their immune signatures.

Thus, the selection of patients to candidate to immunotherapy is still hard. First, we should do clinical and general considerations, such as the evaluation of the treatment goal, the tumour burden and the presence of a symptomatic disease, which might guide the choice depending, for instance, on the need for a rapid response. Then, even if the landmark trials [14,15,16,17,18,21,22,23,24] have shown the existence of a group of patients who benefit more from immunotherapy and maintain the response for a very long time (so-called “responders”), there is still a lack of validated molecular biomarkers that can help in the patients’ selection. In this regard, PD-L1 expression seems to be the most promising biomarker for ICIs. However, PD-L1 is heterogeneously expressed in tumours; additionally, the consensus about the PD-L1 assays, scores (e.g., CPS versus TPS) and cut-offs are still lacking. Thus, starting from PD-L1, other more reliable biomarkers are also needed.

In conclusion, the therapeutic landscape for ESCC is quickly changing. Immunotherapy is becoming an important part of treatment and the continuum of care is not a chimaera in this type of tumour anymore. In this scenario, a multidisciplinary evaluation of the patients is mandatory in order to provide them with proper care that is both the choice of the best and tailored therapeutic strategy/algorithm and simultaneous palliative cares. For instance, the evaluation of nutritional status starting from the diagnosis as well as the palliation of dysphagia or haemorrhages is of huge importance in these patients, requiring dedicated and expert health professionals, mainly working in high volume cancer centres.

However, the journey towards a tailored treatment for those patients is just started and more advances are urgently needed. In this regard, the role of target therapies in addition to immunotherapy, the influence of TME in the response to immunotherapy, the role of ethnicity, the association between immunotherapy and radiotherapy, how to overcome the resistance to immunotherapy, as well as practical manners, such as the best treatment option for elderly people, are unsolved and debated issues today. Finally, further prospective and dedicated trials are still needed in order to find some molecular biomarkers able to predict the response and the prognosis in patients receiving immunotherapy.

## Figures and Tables

**Table 1:** Immune checkpoint inhibitors in the first-line setting for metastatic oesophageal squamous cell carcinoma: landmark phase III and main phase II/III ongoing trials.

Trial/ NCT Number *	Phase	Ethnicity: Asian/Western (%)	Tumour Histology and Primary Site Location	Regimens	Patients (n)	Survival Outcomes/Status *
Landmark trials						
Keynote-590 [14]	III	Asian (53%) Western (47%)	ESCC (73%), EAC (14.8%), GEJA Siewert I (12.1%)	CF CF + pembrolizumab	376 373	Median OS: ESCC and PD-L1 ≥ 10:13.9 vs. 8.8 months, HR: 0.57, *p* < 0.0001 ESCC: 12.6 vs. 9.8 months; HR: 0.72; *p*: 0.0006 PD-L1 ≥ 10:13.5 vs. 9.4 months; HR: 0.62; *p* < 0.0001 entire study population: 12.4 vs. 9.8 months; HR: 0.73; *p* < 0.0001 PD-L1 < 10:10.5 vs. 10.6 months; HR 0·86 median PFS: ESCC: 6.3 vs. 5.8 months, HR: 0.65, *p* < 0.0001; PD-L1 ≥ 10:7.5 vs. 5.5 months, HR: 0.51, *p* < 0.0001; PD-L1 < 10:6.2 vs. 6 months; HR: 0.8; all patients: 6.3 versus 5.8 months, HR: 0.65, *p* < 0.0001.
ESCORT-1 [16]	III	Asian (100%)	ESCC (100%)	C+ paclitaxel C+ paclitaxel + camrelizumab	298 298	Median OS: 15.3 vs. 12.0 months, HR: 0.70, *p* = 0.001 PD-L1 ≥ 1%:HR: 0.59; <1%: HR: 0.79 Median PFS: 6.9 vs. 5.6 months, HR: 0.56, *p*< 0.001 PD-L1 ≥ 1%:HR: 0.51; <1%: HR: 0.62
Checkmate 648 [17]	III	Asian (70%) Western (30%)	ESCC (100%)	CF (a) CF+ nivolumab (b) Nivolumab+ ipilimumab (c)	324 321 325	Median OS: 13.2 (b) versus 12.8 (c) versus 10.7 (a) months PD-L1 ≥ 1%:15.4 (b) versus 13.7 (c) versus 9.1 (a) months PFS: PD-L1 ≥ 1%:HR (b) versus a)): 0.65; HR (c) vs. a)): NR
ORIENT-15 [18]	III	Asian (97.1% Chinese)	ESCC (100%)	CF C+ paclitaxel Chemotherapy+ sintilimab	23 309 327	Median OS: All patients: 16.7 versus 12.5 months, HR: 0.628, *p* < 0.0001 PD-L1 ≥ 10:17.2 versus 13.6 months, HR: 0.638, *p* = 0.0018 Median PFS: All patients: 7.2 versus 5.7 months, HR: 0.558, *p* < 0.0001 PD-L1 ≥ 10:8.3 versus 6.4 months, HR: 0.58, *p* < 0.0001
JUPITER-06 [19]	III	Asian (NR)	ESCC (100%)	C + paclitaxel C + paclitaxel + toripalimab	257 257	Median OS: 17 versus 11 months, HR: 0.58, *p* = 0.00037; Median PFS: HR: 0.58, *p* < 0.00001
Ongoing trials						
LEAP-014 [20] (NCT04949256)	III	Asian and western	ESCC	CF + pembrolizumab CF + pembrolizumab + lenvatinib	NA	Active, recruiting
HERES (NCT05170256)	II	Western	ESCC, HER-2 positive	CF+ pembrolizumab +/− trastuzumab	NA	Active, not yet recruiting
NCT03603756	II	Asian	ESCC	SHR-1210 + apatinib + irinotecan SHR-1210 + apatinib + paclitaxel liposome + nedaplatin	NA	Active, Recruiting
NCT04821765	II	Asian	ESCC, oligometastatic disease	C + nab- paclitaxel+ Tislelizumab + RT→ C + nabpaclitaxel + tislelizumab	NA	Active, Recruiting

* NCT number and state of the trial were shown only for the ongoing trials. Abbreviations: PD-L1: programmed death ligand-1; ESCC: oesophageal squamous cell carcinoma; EAC: Esophageal adenocarcinoma; GEJA: gastroesophageal junctional adenocarcinoma; C: cisplatin; F: 5-fluorouracil; CPS: combined positive score; NE: not evaluable; OS: overall survival; HR: hazard ratio; PFS: progression free survival; TPS: tumour proportion score; NR: not reported; NA: not applicable; RT: radiotherapy.

**Table 3:** Outcomes indirect comparison between immune checkpoint inhibitors in the second-line setting for metastatic oesophageal squamous cell cancer.

Trial	ICI and Target	Median OS *	OS Rate 1-Year (%) *	OS According to PD-L1 Status *	Median PFS *	PFS Rate 1-Year (%) *	ORR *	DCR *
ATTRACTION-3 [21]	Nivolumab (PD-1)	10.9 vs. 8.4 months	47% vs. 34%	PD-L1 TPS ≥1%: 10.9 vs. 8.1 months PD-L1 TPS <1%: 10.9 vs. 9.3 months	1.7 vs. 3.4 months	12% vs. 7%	19% vs. 22%	37% vs. 63%
Keynote-181 ** [22]	Pembrolizumab (PD-1)	8.2 vs. 7.1 months	39.4% vs. 24.9%	PD-L1 CPS ≥10: 10.3 vs. 6.7 months	2.2 vs. 3.1 months	15.3 vs. 9.4%	16.7% vs. 7.4%	42.9% vs. 49.7%
ESCORT [23]	Camrelizumab (PD-1)	8.3 vs. 6.2 months	34% vs. 22%	PD-L1 TPS ≥1%: 9.2 vs. 6.3 months	1.9 vs. 1.9 months	10% vs. NA	20.2% vs. 6.4%	44.7% vs. 34.5%
RATIONAL 302 [24]	Tislelizumab (PD-1)	8.6 vs. 6.3 months	37% vs. 24%	PD-L1 vCPS: 10.3 vs. 6.8 months	NR	NR	20.3% vs. 9.8%	NR

* all the comparisons are referred to the control arm (chemotherapy); ** results reported for the ESCC cohort only. Abbreviations: ICI: immune checkpoint inhibitor; OS: overall survival; PD-L1: programmed death ligand-1; PFS: progression-free survival; ORR: overall response rate; DCR: disease control rate; PD-1: programmed death-1; vs.: versus; NA: not available; NR: not reported; TPS: tumour proportion score; CPS: combined positive score; vCPS: visual combined positive score.

**Table 4:** PD-L1 assessment and expression in oesophageal squamous cell carcinoma treated within landmark immunotherapy trials.

Trial	Line of Treatment	Selection by PD-L1	PD-L1 Expression Test and Score	PD-L1 Cut off	PD-L1 Patients %
Keynote-590 [14]	1st	unselected	IHC (available on 97.5% of patients) CPS	≥10	Pos: 51.1% Neg: 46.3%
ESCORT-1 [16]	1st	unselected	IHC (available on 98.2% of patients) TPS	≥1%	pos: 55.2% neg: 42.9%
Checkmate 648 [17]	1st	unselected	IHC (available on 99.5% of patients) TPS CPS (preplanned)	≥1%	Pos: 49% Neg: 50.7%
ORIENT-15 [18]	1st	unselected	NR TPS and CPS	≥10% on TPS ≥10 on CPS	Pos: TPS ≥ 10%: 36.1% CPS ≥ 10%: 57.8% Neg: NR
JUPITER-06 [19]	1st	unselected	IHC (available on 94.9% of patients) CPS	≥1	Pos: 78% Neg: 16.9%
ATTRACTION-3 [21]	2nd	unselected	IHC (available on 94.9% of patients) TPS	≥1%	Pos: 48.5% Neg: 51.5%
Keynote-181 [22]	2nd	unselected	IHC (available on 98.6% of patients) CPS	≥10	Pos: 35.35% Neg: 63.2%
ESCORT [23]	2nd	unselected	IHC (available on 100% of patients) TPS	≥1%	Pos: 44.9% Neg: 55.1%
RATIONAL 302 [24]	2nd	unselected	IHC (available on % of patients: NR) vCPS	≥10	Pos: 30.6% Neg: 63.2%

Abbreviations: PD-L1: programmed death ligand 1; IHC: immunohistochemistry; pos: positive; neg: negative; TPS: tumour proportional score; CPS: combined positive score; vCPS: visual combined positive score; NR: not reported.
